# HAARN: A Deep Neural Network-Based Intelligent Control Method for High-Altitude Adaptability of Heavy-Load UAV Power Systems

**DOI:** 10.3390/s26020389

**Published:** 2026-01-07

**Authors:** Haihong Zhou, Xinsheng Duan, Xiaojun Li, Jianrong Luo, Bin Zhang, Xiaoyu Guo, Lejia Sun

**Affiliations:** 1 Shaanxi Power Transmission and Transformation Engineering Company Limited, Xi’an 710003, China; haihongzhoushaanxipower@gmail.com (H.Z.); xinshengduanshannxipower@hotmail.com (X.D.); xiaojunlishannxipower@hotmail.com (X.L.); jianrongluoshannxipower@hotmail.com (J.L.); xiaoyuguoshannxipower@hotmail.com (X.G.); 2 State Grid Shaanxi Electric Power Co., Ltd., Construction Branch, Xi’an 710005, China; binzhangshaanxielectric@hotmail.com; 3 School of Microelectronics, Xidian University, Xi’an 710126, China

**Keywords:** heavy-load UAV, ultra-high voltage (UHV) construction, high-altitude adaptability, deep neural network (DNN), power system regulation

## Abstract

The construction of ultra-high voltage transmission lines puts extremely high demands on the high-altitude operation of heavy-load unmanned aerial vehicles (UAV). Air density and temperature at high altitudes have a great influence on the efficiency and stability of the UAV power system. Traditional regulation methods based on parameters pre-set or simple look-up tables cannot achieve the best adaptability. In this paper, we presents an intelligent method for the high-altitude adaptability control of heavy-load UAV power systems using a deep neural network. The proposed method collects real-time, multi-dimensional environmental parameters, including altitude, temperature, and air pressure, using a barometric altimeter and GPS receiver, constructs a High-Altitude Adaptive Regulation Network (HAARN), and intelligently learns complex nonlinear relationships to predict the optimal motor speed, propeller pitch angle, and current limit under the current environmental conditions so as to dynamically adjust power output. The HAARN model was trained on a dataset of 12,000 synchronized samples collected from both controlled environmental-chamber experiments (temperature range: −10 °C to 20 °C; pressure range: 100–50 kPa, corresponding approximately to 0–5500 m) and multi-point plateau flight trials conducted at 2000 m, 3000 m, 4000 m, and 4500 m. This combined dataset was used for feature engineering, exhaustive-label generation, and model validation to ensure robust generalization across realistic high-altitude operating conditions. Experimental results show that, compared with traditional PID control and lookup-table approaches, the proposed method reduces thrust attenuation by about 12.5% and improves energy efficiency by 8.3% at the altitude of 4000 m. In addition, HAARN demonstrates consistent improvements across the tested altitude range (0–4500 m).

## 1. Introduction

Ultra-high voltage (UHV) transmission lines are crucial infrastructure for national energy strategies and major engineering projects such as the “West-to-East Power Transmission” program [1,2,3]. These lines often traverse high mountains, canyons, and deserts, creating harsh construction environments that increase the need for high-altitude heavy-load UAV operations [4,5]. HD-UAVs, with strong load-carrying capacity and flexible deployment advantages, have enormous potential in material delivery, conductor pulling, and high-precision inspection [6,7,8]. They have become indispensable key technical equipment in UHV construction. However, severe challenges were posed to UAV power systems by rarefied air and low-temperature environments in high-altitude areas. With increased altitude, there was a sharp decrease in air density ρ and ambient temperature, directly influencing the’ lift force of the rotors and the’ heat dissipation performance of the motors: thrust attenuation limits the maximum load and safety margin, while deteriorated thermal management easily overheats motors and Electronic Speed Controllers (ESCs) [9]. This may lead to system degradation or even shutdown risks, seriously endangering mission safety. Until now, existing methods have mainly addressed these issues by increasing redundant power or adopting conservative flight strategies, but they significantly reduce endurance and energy efficiency. Thus, developing an intelligent method that enables adaptive regulation and energy consumption optimization of power systems in complex plateau environments has become a key direction to enhance the unmanned construction capability of UHV projects.

The commonly used traditional regulation methods for the power system of UAVs mainly fall into three categories: first, attitude control based on PID [10,11,12,13,14,15], which can maintain the stability of flight but cannot dynamically respond to the influence of altitude variation on thrust characteristics; second, compensation methods [16,17] using the ISA model depend only on theoretical calculations without considering the interference from local outdoor temperature and humidity, which leads to insufficient accuracy and robustness; third, the Lookup Table approach [18,19] conducts power correction based on preset discrete compensation coefficients; yet, the interpolation accuracy and data dimensions will constrain it, which cannot work well for the nonlinear coupling of many influence factors such as altitude, temperature, and humidity. In recent years, with the rapid development of AI and computer technology, machine learning has been gradually applied in adaptive optimization for UAVs [20,21,22,23,24]. However, existing studies generally focus on single-parameter regulation, lacking overall modeling and joint optimization for environmental factors, ETE, and thermal management constraints, which makes it hard to meet the complex requirements of HD-UAV plateau missions.

To address the above issues, this paper proposes a High-Altitude Adaptive Intelligent Regulation Network (HAARN) for HD-UAV power systems based on Deep Neural Networks (DNN). The main contributions of this paper are summarized as follows:

1. Multi-sensor fusion-based high-precision environmental perception: Data fusion of barometric altimeters and GPS locators is realized, providing stable and high-precision real-time multi-dimensional environmental feature inputs (such as altitude, ambient temperature, air pressure) for HAARN.

2. End-to-end deep learning regulation architecture: A multi-layer HAARN model is built which can learn intelligently the complex nonlinear mapping relationship between the environmental parameters and optimal control commands (rotational speed, propeller pitch angle, current limit).

3. Synergistic optimization of energy consumption and reliability: With maximum thrust per unit of energy consumption (ETE) as the primary optimization objective and the output of current limits at the same time, this achieves the synergy in thrust compensation and thermal protection to improve reliability and endurance for plateau operations.

The remainder of this paper is organized as follows. Section 2 analyzes the quantitative impact of high-altitude environments on UAV power systems and discusses limitations of traditional regulation methods. Section 3 presents the proposed HAARN framework, including multi-sensor fusion, dataset construction, network architecture, and optimization objectives. Section 4 details the experimental setup and data-collection procedures, and Section 5 presents experimental results and analysis, including static and dynamic performance comparisons, energy-efficiency evaluation, and thermal reliability tests. Finally, Section 6 concludes the paper and outlines directions for future work.

## 2. Analysis of Adaptability Issues of UAV Power Systems in High-Altitude Environments

### 2.1. Quantitative Impact of Plateau Environments on UAV Dynamic Performance

The fundamental cause of thrust attenuation in UAVs operating in plateau environments is the reduction in air density [25]. In the International Standard Atmosphere (ISA) model, the variation of air density ρ with altitude *H* (unit: m) can be approximately expressed as

(1) $$\rho \left(H\right)=\rho_{0}{\left(1-\frac{\lambda H}{T_{0}}\right)}^{\frac{g}{\lambda R}-1}$$

where $\rho_{0}$ is the standard air density at sea level (1.225 kg/m^3^), $T_{0}$ is the standard temperature at sea level (288.15 K), λ is the temperature lapse rate (0.0065 K/m), *g* is the gravitational acceleration and *R* is the gas constant.

According to the thrust formula T∝ρ, the thrust attenuation rate $\eta_{T}$ can be defined as follows:

$$\eta_{T}=\frac{T(0)-T(H)}{T(0)}=1-\frac{\rho (H)}{\rho_{0}}$$

At an altitude of 4000 m, the thrust loss typically exceeds 35.

$$\omega_{comp}=\omega_{req}\sqrt{\frac{\rho_{0}}{\rho (H)}}$$

The increase in rotational speed leads to a significant increase in motor input current *I* and energy consumption *P*, exacerbating the challenges in energy efficiency of the system [26,27,28].

### 2.2. Thermal Management and Reliability Analysis in Plateau Environments

The low-pressure environment in high-altitude areas not only affects thrust but also severely impairs the thermal management performance of motors and Electronic Speed Controllers (ESCs). The loss of motor power $P_{loss}$ is mainly converted into heat and its heat dissipation $Q_{diss}$ is achieved primarily by thermal convection. In a low-pressure and low-density high-altitude environment, the convective heat transfer coefficient *h* decreases, resulting in an increase in thermal resistance $R_{th}$.

$$R_{th}=\frac{1}{hA_{surf}}$$

where $A_{surf}$ is the motor surface area. The steady-state motor temperature $T_{motor}$ can be approximated as follows:

$$T_{motor}=T_{env}+P_{loss}\;\cdot \;R_{th}$$

Due to the increase in $P_{loss}$ caused by thrust compensation and the simultaneous increase in $R_{th}$, the final $T_{motor}$ increases significantly. If $T_{motor}$ exceeds the winding insulation class or the maximum operating temperature of the ESC, the system will trigger thermal protection or suffer permanent damage. Traditional controllers lack the coupled perception of ambient temperature $T_{env}$ and real-time motor temperature, making it impossible to dynamically adjust the power output limit and thus failing to ensure system reliability under extreme operating conditions.

**Figure 1:**
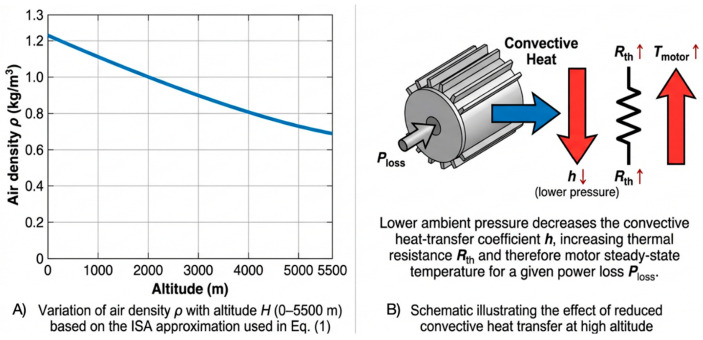
(**A**) Variation of air density ρ with altitude H (0–5500 m) based on the ISA approximation used in Equation (1). The plot visually demonstrates the rapid decrease of air density at plateau altitudes and supports the following discussion of thrust attenuation. (**B**) Schematic illustrating the effect of reduced convective heat transfer at high altitude: lower ambient pressure decreases the convective heat-transfer coefficient h, increasing thermal resistance $R_{th}$ and therefore motor steady-state temperature for a given power loss $P_{loss}$.

To illustrate these effects, Figure 1A plots the ISA-based air-density variation versus altitude, and Figure 1B provides a schematic of the resulting increase in thermal resistance and motor temperature.

### 2.3. Limitations of Mathematical Models in Traditional Regulation Mechanisms

The output u(t) of a traditional PID controller is:

$$u\left(t\right)=K_{p}e\left(t\right)+K_{i}\int_{0}^{t}e\left(\tau \right)d\tau +K_{d}\frac{de(t)}{dt}$$

In high-altitude environments, the thrust response function of the system $G_{T}\left(s\right)$ changes: $G_{T}\left(s\right)\to G_{T}^{\prime }\left(s\right)$. Continuing to use the $K_{p},\;K_{i},\;K_{d}$ calibrated at sea level may lead to a deteriorated dynamic system response (e.g., oscillation, overshoot) or even instability. The regulation based on the Lookup Table method $u_{LT}\left(H\right)$ is only:

$$u_{LT}\left(H\right)=f_{LT}\left(H\right)\;\cdot \;u_{nominal}$$

This method is a univariate mapping that does not reflect the impact of ambient temperature $T_{env}$ on motor winding resistance $R_{winding}$ ($R_{winding}\propto T_{motor}$), thus unable to achieve refined regulation of energy efficiency and thermal limits.

## 3. Intelligent Regulation Method of Power System Based on Deep Neural Network

### 3.1. Multi-Sensor Data Fusion Algorithm

To provide high-precision and high-reliability real-time altitude information, we adopts the Extended Kalman Filter (EKF) algorithm to fuse data from the barometric altimeter ($H_{bar}$) and GPS locator ($H_{gps}$).

The barometric altimeter provides high-bandwidth and low-drift relative altitude information but is susceptible to meteorological changes. GPS offers accurate absolute altitude information but has a low update frequency and occasional jumps. EKF leverages the complementarity of the two to achieve optimal state estimation.

The state variable x is defined as:

$$x_{k}={\left[H_{k},\;v_{k},\;a_{k}\right]}^{T}$$

where $H_{k}$ is the estimated altitude at time *k*, $v_{k}$ is the vertical velocity, and $a_{k}$ is the vertical acceleration.

The state prediction model $f(x_{k-1})$ adopts a uniform acceleration model for prediction:

$$x_{k}=Fx_{k-1}+w_{k}$$

where the state transition matrix F is modified to include the acceleration term:

$$F=\left(\begin{array}{ccc} 1 & \Delta t & 0.5\Delta t^{2}\\ 0 & 1 & \Delta t\\ 0 & 0 & 1 \end{array}\right)$$

Δt is the sampling time interval, $w_{k}$ is the noise of the process, and its covariance Q is adjusted according to the kinematic characteristics of the UAV in the plateau. The diagonal elements of Q reflect the model’s confidence in the uncertainty of $H_{k},\;v_{k},\;a_{k}$, which are usually increased under high wind speed conditions in the plateau.

The observation variable z is defined as:

$$z_{k}={\left[H_{bar,k},H_{gps,k}\right]}^{T}$$

The observation model is $z_{k}=Hx_{k}+v_{k}$, and the observation matrix H is:

$$H=\left(\begin{array}{ccc} 1 & 0 & 0\\ 1 & 0 & 0 \end{array}\right)$$

$v_{k}$ is the observation noise and its covariance matrix $R=\mathrm{diag}(\sigma_{bar}^{2},\sigma_{gps}^{2})$. In practical applications, we dynamically adjust $\sigma_{gps}^{2}$ based on GPS signal quality (HDOP value) to reduce its weight when the GPS signal is poor, achieving robust fusion. The final output is a stable fused altitude estimate $H_{fusion}$ with a resolution better than 0.5 m.

### 3.2. Data Collection and Feature Engineering

**Figure 2:**
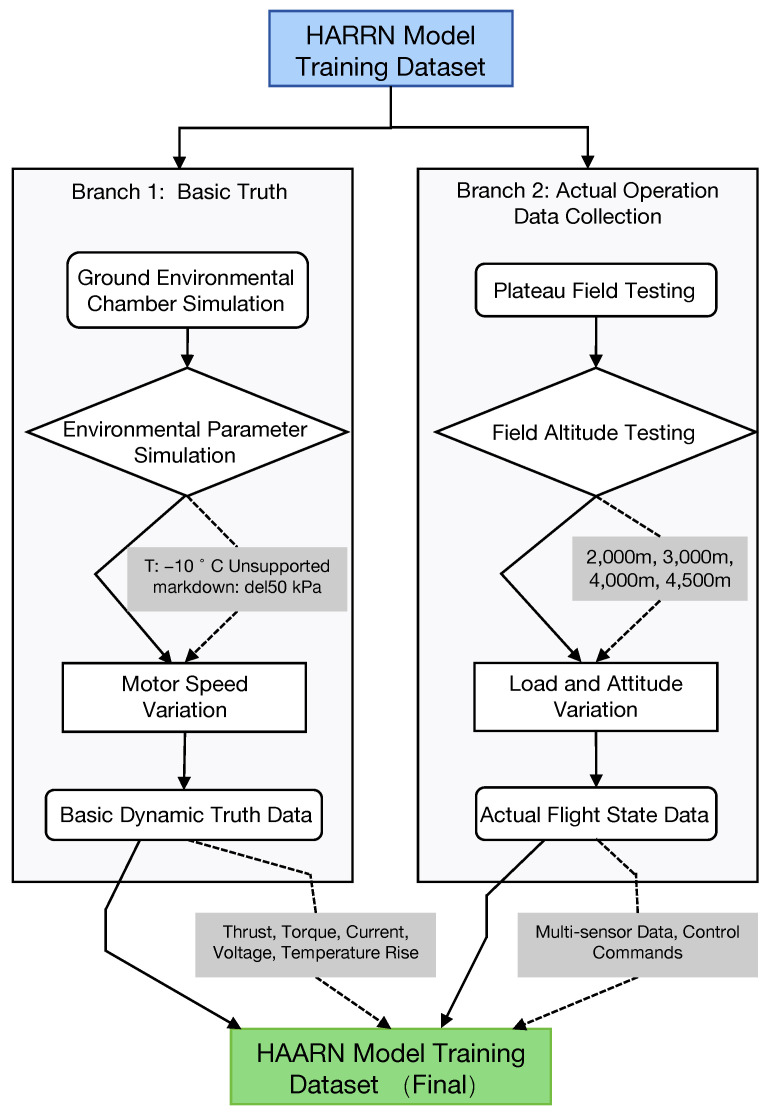
An overview of data collection and dataset construction. The workflow is divided into simulated “Basic Truth” (Branch 1) and “Actual Operation” (Branch 2). Solid arrows indicate the procedural flow, while dotted arrows denote specific data parameters or environmental constraints (e.g., pressure, altitude, and sensor outputs). Colors highlight the initial dataset (blue), processing stages (white), and the final consolidated training dataset (green).

The training dataset of the HAARN model is the foundation for achieving accurate regulation. As illustrated in Figure 2, data collection follows strict experimental protocols, covering all key operating quadrants in plateau environments. The establishment of data sources and collection environments combines two methods: first, in a ground environmental chamber, we simulated a temperature range of −10 °C to 20 °C and an air pressure range of 100 kPa to 50 kPa (corresponding to altitudes from 0 m to 5500 m). Real-time thrust, torque, current, voltage, and temperature rise data of the motor at different rotational speeds were accurately measured in the chamber to construct a basic dynamic truth database. Second, we conducted plateau field tests with varying loads (20 kg to 50 kg) and attitude changes (±15° tilt) at altitudes of 2000 m, 3000 m, 4000 m, and 4500 m, collecting multi-sensor data and control commands under actual flight conditions.

The generation of true value labels $Y_{true}$ follows the criterion of maximizing the thrust per unit of energy consumption (ETE) while considering the safe temperature limit of the motor. The true values of $\omega_{opt}$ and $\delta_{pitch}$ in $Y_{true}={\left[\omega_{opt},\delta_{pitch},I_{limit}\right]}_{true}^{T}$ are obtained, for each environmental state (a subset of X), through an exhaustive search and an inverse solution of the dynamic model to find the combination of ω and $\delta_{pitch}$ that can provide the required thrust $T_{req}$ and maximize ETE=T/(V·I). Meanwhile, the $I_{limit,true}$ label is the theoretical safe current upper limit calculated based on the maximum allowable temperature rise $T_{max,safe}$ and the thermal balance model under that state.

In the feature preprocessing stage, all sensor data are aligned and synchronized using high-precision timestamps and downsampled to a uniform frequency of 20Hz. Subsequently, all input features X are assigned to the [0,1] interval using the Min-Max normalization method. The denormalization of the output Y is completed before flight control execution to ensure the output commands are within the physical safety range. The input feature set X of the HAARN model has 7 dimensions, including $H_{fusion}$, $T_{env}$, $P_{abs}$, RH, $I_{motor}$, $\omega_{motor}$, and the newly added $T_{surface}$ (motor surface temperature), thus achieving a comprehensive perception of the environment, dynamic state, and thermal state.

### 3.3. Construction of High-Altitude Adaptive Regulation Network (HAARN) Model

**Figure 3:**
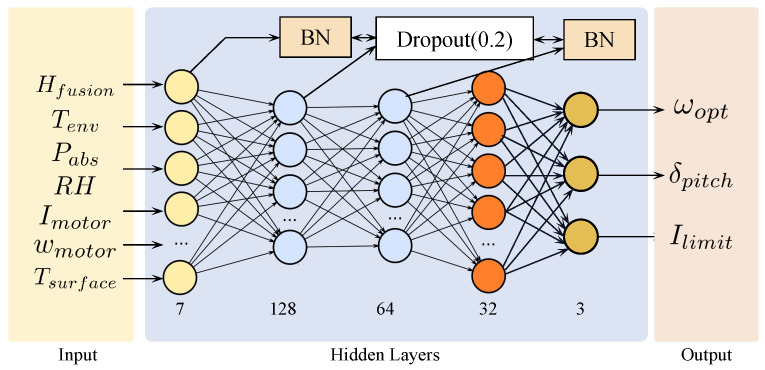
Overview of the High-Altitude Adaptive Regulation Network (HAARN). It is a lightweight 4-layer fully-connected network (node structure: 7-128-64-32-3) with Batch Normalization (BN) and Dropout (p=0.2) for stability and generalization. Inputs: $H_{\mathrm{fusion}}$ (EKF-fused altitude, ≤0.5 m), $T_{\mathrm{env}}$ (ambient temp), $P_{\mathrm{abs}}$ (absolute pressure), RH (relative humidity), $I_{\mathrm{motor}}$ (motor current), $\omega_{\mathrm{motor}}$ (motor speed), $T_{\mathrm{surface}}$ (motor surface temp). Outputs: $\omega_{\mathrm{opt}}$ (optimal motor speed for PID throttle compensation), $\delta_{\mathrm{pitch}}$ (optimal propeller pitch for actuator control), and $I_{\mathrm{limit}}$ (safe current limit for ESC dynamic restriction).

HAARN is a lightweight, four-layer fully connected network that minimizes model complexity while guaranteeing high-precision nonlinear mapping performance, with the requirement to meet the resource constraints and real-time demand for flight control computing units. The network topology is selected as (7-128-64-32-3). In that sense, it makes a good balance between accuracy and latency. Specifically, from the input layer (7 nodes) to the first hidden layer (128 nodes), mapping low-dimensional environmental features onto a high-dimensional feature space captures complex environmental coupling relationships, and then two subsequent hidden layers are used to extract, compress, and refine high-dimensional features for the mapping function learning to transform the environmental features into optimal control strategies. The detailed architecture of the network is illustrated in Figure 3.

Specifically, BN is applied after Hidden Layer 1 and Hidden Layer 3 to stabilize the internal covariance shift of the network at first. This ensures stable training and fast convergence of the model under significantly different data distributions (Domain Shift) collected at high altitudes and sea levels. Second, a Dropout layer with p=0.2 in Hidden Layer 2 serves as a regularization method to reduce co-adaptation between neurons, enhancing generalization capability in unknown or transiently changing plateau meteorological conditions. Finally, for the selection of the activation function, ReLU is adopted for all hidden layers to guaranty computational efficiency. In the output layer, $\omega_{opt}$ and $\delta_{pitch}$ employ linear activation functions to allow free adjustment within the target range; for $I_{limit}$, the Sigmoid activation function is adopted, followed by scaling and translation operations to strictly limit the current between the upper and lower limits of the safe operating current of the motor $I_{min}\leq I_{limit}\leq I_{max}$ for physical constraints. It is worth noting that the generalization across different UAV platforms mainly requires adjusting the scaling ranges of motor–propeller parameters, while the core HAARN structure remains unchanged.

### 3.4. Optimization Objective and Loss Function Improvement

The training objective of HAARN is based on maximizing energy efficiency while taking thermal reliability as an insurmountable constraint. The final hybrid loss function adopted $L=L_{ETE}+\lambda_{H}L_{Heat}$ achieves synergistic optimization of energy efficiency and reliability. Among them, the thrust energy efficiency loss term $L_{ETE}$ uses the standard Mean Squared Error (MSE) as the main optimization term:

$$L_{ETE}=\frac{1}{N}\sum_{i=1}^{N}\left|\right|Y_{pred,i}-Y_{true,i}{\left|\right|}^{2}$$

By minimizing this loss, the network’s predicted control commands are prompted to approach the maximum true ETE values at each environmental point to the greatest extent. Meanwhile, to actively manage thermal risks, a dynamic thermal limit and penalty term $L_{Heat}$ are introduced. We established a first-order thermal model based on the equivalent heat capacity and thermal resistance of the motor windings to dynamically calculate the maximum safe current $I_{safe}$ during training. This model is based on the thermal balance equation: $\frac{dT_{motor}}{dt}=\frac{1}{C_{th}}\left(P_{loss}-Q_{diss}\right)$. Based on this model and the $T_{max,safe}$ threshold, we dynamically calculate $I_{safe}$ and construct the penalty term:

$$L_{Heat}=\lambda_{H}\;\cdot \;\frac{1}{N}\sum_{i=1}^{N}\max{\left(0,I_{limit,pred}-I_{safe,model}\right)}^{2}$$

where $I_{limit,pred}$ is the current limit in the output $Y_{pred}$. The weight coefficient $\lambda_{H}$ is set to a high value to assign a higher priority to optimization to thermal limits, ensuring that thrust compensation does not come at the cost of motor life. It should be noted that the thermal model used in $L_{Heat}$ is calibrated under controlled temperature–pressure conditions and is mainly designed as a conservative constraint; therefore, it does not explicitly target highly transient or extreme meteorological variations.

### 3.5. Flight Control Platform Deployment of Intelligent Regulation Strategy

Real-time deployment of the HAARN model is essential to ensure stable UAV operation in plateau environments. The model is deployed directly on the onboard flight-control computing unit (e.g., a high-frequency MCU integrated into the main control board), rather than on external edge-computing devices, to guarantee compactness, robustness, and deterministic real-time behavior.

To satisfy the hard real-time constraints of the flight-control loop, the trained HAARN model undergoes strict post-training optimization before deployment. All network weights and activations are quantized from 32-bit floating-point (FP32) to 8-bit fixed-point (INT8), reducing the model size by approximately 75% and yielding a 3–5× inference speedup compared with the FP32 implementation. The quantized model is compiled with architecture-specific optimizations targeting ARM Cortex-M7/H7 microcontrollers, enabling efficient execution without dedicated neural acceleration hardware. After optimization, the INT8-quantized HAARN model occupies approximately 28 kB of RAM for weights and intermediate buffers, and 48 kB of flash memory for the model binary. On a representative flight-control computing unit (ARM Cortex-M7@400 MHz), the average per-inference execution time, including feature normalization and output denormalization, is 8–10 ms. The target flight-control computing unit is based on an STM32H7-series microcontroller (STMicroelectronics, Geneva, Switzerland), featuring an ARM Cortex-M7 core running at 400 MHz.

The HAARN module is integrated into the main flight-control loop and executed at 20 Hz, corresponding to a 50 ms control-cycle budget. At the perception stage, the Extended Kalman Filter (EKF) outputs the fused altitude-related state $H_{\mathrm{fusion}}$ together with six additional environmental and flight-state features X. These features are processed by the onboard computing unit, and HAARN completes inference within ≤10 ms to generate the regulation output $Y_{pred}$. The observed end-to-end latency from EKF output to the updated Electronic Speed Controller (ESC) current limit $I_{limit}$ does not exceed 12 ms. When operating at 20 Hz, HAARN inference accounts for approximately 4–8% of the available CPU cycles on the test MCU, leaving more than 90% computational headroom for other time-critical modules, including EKF, PID control, and sensor fusion. Stack and heap usage during inference remain well within the available memory budget of the flight-control board.

At the execution stage, the predicted thrust regulation commands $\omega_{opt}$ and $\delta_{pitch}$ are applied as feedforward compensation signals and superimposed on the baseline PID controller output. In parallel, the predicted current limit $I_{limit}$ is transmitted to the ESC as a dynamic upper bound. When the motor current approaches this limit, the ESC softly constrains power output, enabling proactive thermal protection without relying solely on delayed temperature sensor feedback. Importantly, HAARN operates as an auxiliary feedforward module rather than a replacement for the core stabilization loop. The PID controller remains the primary safety-critical component, and in the presence of abnormal predictions, timing overruns, or sensor inconsistencies, the system automatically disables HAARN and reverts to pure PID control, thereby preserving deterministic flight-control behavior and ensuring operational safety.

## 4. Experimental Setup and Data Preparation

This section describes the experimental platform, sensor configuration and calibration procedures, environmental-chamber protocols, plateau flight-test sites and procedures, data acquisition and synchronization methods, and dataset statistics used for HAARN training and evaluation. These details are provided to ensure reproducibility and to clarify how ground-truth labels and input features were obtained.

### 4.1. Experimental Platform and Data Preparation

#### 4.1.1. Experimental Platform

We conducted experiments using a customized heavy-duty hexacopter UAV platform, whose maximum takeoff weight is 50 kg. It is equipped with variable-pitch carbon fiber propellers (32 inches in diameter) to fully utilize the $\delta_{pitch}$ commands output by HAARN. The power system consists of a high-power BLDC with a KV value of 120, powered by a 12S 22,000 mAh lithium-polymer battery pack. The flight control system is equipped with a high-performance flight control computing unit which has enough processing capacity to run the quantized HAARN model. For each environmental-condition point used in the dataset, at least three independent repetitions were performed to quantify measurement dispersion. Reported metrics include sample standard deviations and confidence intervals where applicable; uncertainty sources such as sensor noise, mounting tolerances, and environmental fluctuations.

#### 4.1.2. Sensor Calibration and Configuration

In particular, we carried out an accurate sensor configuration to meet the 7-dimensional input features of HAARN. Environmental perception utilized a high-precision MS5611 barometric altimeter (TE Connectivity, Schaffhausen, Switzerland) and a u-blox M8N GPS module (u-blox AG, Thalwil, Switzerland) for EKF fusion as in Section 3.1, the output of which is $H_{fusion}$, while an ambient temperature sensor $T_{env}$ and a humidity sensor RH were equipped. In terms of perception of the state of the power system, in addition to the real-time current $I_{motor}$ and the rotational speed $\omega_{motor}$ fed back by the ESC, a high-temperature resistance sensor PT1000 (Heraeus Nexensos, Hanau, Germany) was deployed near the motor winding housing for accurate monitoring of the motor surface temperature $T_{surface}$, which directly informed the thermal management process.

Prior to data collection, all sensors underwent calibration. The MS5611 barometer was zero-offset calibrated using a precision reference barometer (Vaisala Oyj, Vantaa, Finland), and its bias corrected; the u-bloxUBLOX M8N GPS module (u-blox AG, Thalwil, Switzerland) was verified for altitude accuracy using differential GPS in a static test (HDOP threshold set to <2.0 to accept samples); the PT1000 temperature sensor was calibrated against a NIST-traceable thermometer (Fluke Calibration, Everett, WA, USA) with an uncertainty of ±0.1 °C). ESC-reported motor speed $\omega_{motor}$ and motor-current $I_{motor}$ were validated against an independent optical tachometer (UNI-T, Dongguan, China) and a Hall-effect current sensor (LEM Group, Geneva, Switzerland), respectively. Sensor time-stamping was synchronized using the flight control’s hardware clock; all data streams were aligned to within ±1 ms.

#### 4.1.3. Data Collection Environment

The experimental data were gathered in two phases: In the first phase, all static envelope tests with temperatures from −10°C to 20°C and air pressures from 100 kPa to 50 kPa were completed in the ground environmental chamber for accurate calibration and building of HAARN’s truth database. The second phase consisted of multi-point field flight tests, which were performed at 2000 m, 3000 m, 4000 m, and 4500 m altitude over the Qinling-Mountains area, with operating conditions including fixed-height hovering, vertical climbing, and extreme load transportation. All the data were collected synchronously at a frequency of 50 Hz; 12,000 valid data samples were obtained, of which 10,000 were used for training and 2000 for validation and final testing.

As shown in Figure 5, in the environmental-chamber tests, thrust ground-truth was obtained using a six-axis load cell (resolution 0.1 N, accuracy ±0.5%) rigidly mounted to a static test fixture. The environmental chamber controlled temperature and pressure with accuracies of ±0.5 °C and ±0.5 kPa, respectively. Each static operating point in the chamber was measured for 60 s and repeated three times; transient tests were repeated five times to capture variability. During plateau field tests, thrust estimates were cross-validated with in-situ load-cell bench tests and corrected for mounting effects. Battery state-of-charge (SOC) was maintained between 70 ± 5% in all flight tests to minimize power-supply variability; voltage and SOC were logged continuously and used to filter out samples outside the specified range.

## 5. Experimental Results and Analysis

In this section, we present the experimental results. Subsections include: static regulation performance comparisons (steady hover), dynamic thrust step-response analysis, energy-efficiency (ETE) comparisons across altitudes, and thermal-reliability assessments. Each experiment is reported with measurement details, uncertainty quantification, and comparison against baseline methods.

### 5.1. Static Comparison Experiment of Regulation Performance

To assess the steady-state performance of HAARN in suppressing plateau thrust attenuation, three fixed-height hovering tests were conducted in a typical plateau environment with altitude H=4000 m and ambient temperature T=10°C, maintaining constant thrust $T_{req}$ required by heavy-duty tasks. Three different regulation methods were compared: the traditional PID control based on sea-level parameters, a simple lookup table method modified by the ISA model, and the proposed HAARN method.

#### Core Performance Indicators

In addition to the traditional thrust loss rate, we introduced the Regulation Precision Index (RPI), defined as the Root Mean Square Error (RMSE) of the deviation between the target thrust and the actual thrust. Meanwhile, the EKF-fused altitude estimate $H_{fusion}$ was independently verified to ensure the reliability of the input of HAARN’s. In the 4000-m test, the RMSE of $H_{fusion}$ after EKF fusion was reduced by 68% compared to the standalone GPS altimeter.

As shown in Table 1, due to the lack of prior knowledge about changes in thrust characteristics, traditional PID control has insufficient thrust compensation, with the thrust loss rate as high as 15.0%. A simple lookup table method reduces this to 9.8% by simple density compensation. In contrast, the HAARN method has an extremely low thrust loss rate of only 2.5%, realizing a quasi-sea-level thrust output. Meanwhile, in optimal thrust compensation performance, HAARN has a slightly lower average motor speed compared to the lookup table method, while consuming less current, 13.0 A versus 14.2 A. This shows that HAARN successfully utilizes the regulatory effect of the propeller pitch angle $\delta_{pitch}$ to obtain thrust by optimizing aerodynamic efficiency without relying on higher rotational speeds, avoiding unnecessary increases in energy consumption. Its regulation response time is only 40 ms, which is 40% that of the lookup table method, reflecting the real-time decision-making advantages of the deep learning model.

### 5.2. Dynamic Characteristic Analysis of Thrust Response

The thrust step response test was performed to assess the adaptability of the control system to sudden changes in the external environment and flight commands. In the experiment, at an altitude of 4000 m, the system inputted a step command from hovering thrust to 80% of maximum thrust. Then, the dynamic characteristics of the thrust output were analyzed. The important indicators of dynamic performance include Rise Time, settlement Time, and Overshoot.

#### 5.2.1. Thrust Step Response Curve Comparison

**Figure 4:**
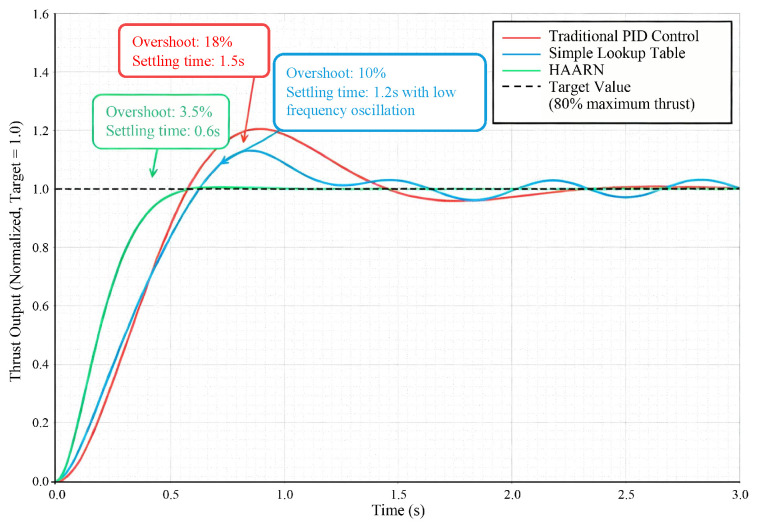
Comparison of thrust step response curves (4000 m altitude).

Figure 4 clearly illustrates the comparative dynamic performance of the three methods. Due to its system gain variation due to high-altitude thrust characteristics, the conventional PID demonstrates 18% overshoot and a settling time of as long as 1.5 s, which will inevitably lead to a severe oscillation and instability of the attitude during actual flight. The simple lookup table method, although by compensation reduces the overshoot to 10%, still possesses a low-frequency oscillation of about 3% in the process of stabilization, due to the discreteness of its compensation coefficients. The HAARN method has an overshoot of only 3.5% and a significantly shortened settling time of 0.6 s. This excellent dynamic performance is directly attributed to the thrust feedforward compensation mechanism described in Section 3.5: The optimal commands predicted in real-time $\omega_{opt}$ and $\delta_{pitch}$ by HAARN are used directly as feedforward signals to correct the throttle command, reducing the dependence of the underlying PID controller on errors. This allows the system to predict in advance and adjust quickly, guaranteeing the attitude stability and safety of the heavy-duty UAV while going through complex airflow environments. The quantitative comparison of key dynamic performance indicators at 4000 m altitude is summarized in Table 2.

#### 5.2.2. Thrust Response to 80% Step Command at 4000 m

**Figure 5:**
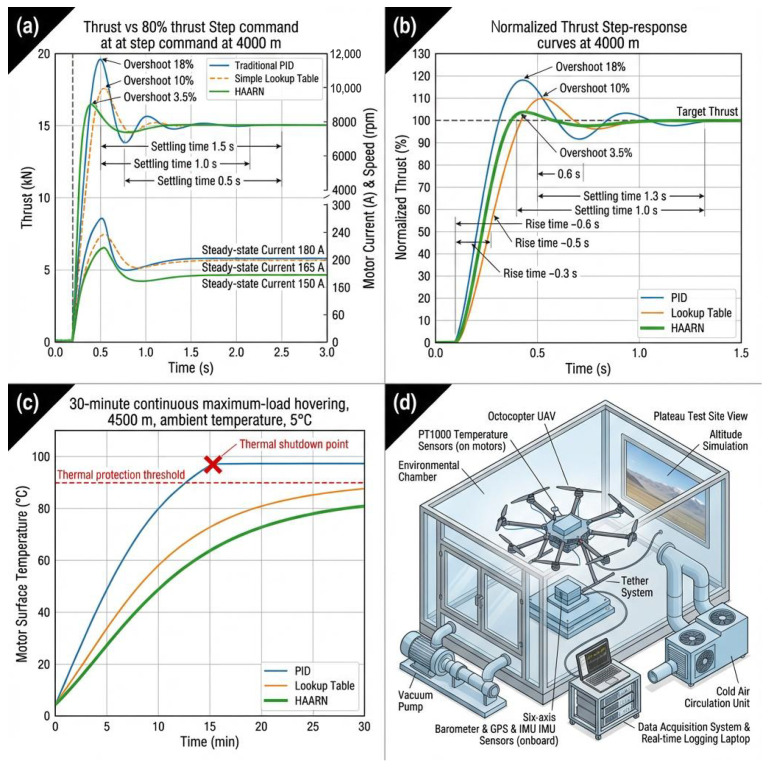
Comparison of thrust regulation methods (PID, lookup-table, and HAARN) under high-altitude conditions. (**a**) Time-series responses of thrust, motor current, and speed for an 80% step at 4000 m, showing reduced overshoot and lower steady-state current with HAARN. (**b**) Normalized thrust step responses at 4000 m with annotated overshoot, rise time, and settling time. (**c**) Motor surface temperature during 30-min maximum-load hovering at 4500 m, where HAARN remains below the thermal limit while PID triggers protection. (**d**) Environmental-chamber and plateau flight-test setup, including load cell, PT1000 sensors, and data acquisition system.

Figure 5a compares time-series of thrust, motor current, and speed for an 80% step thrust test at 4000 m using PID (blue), lookup table (orange), and HAARN (green). HAARN shows 3.5% overshoot (vs. 10% and 18%), 0.5 s settling time (vs. 1.0 s and 1.5 s), and lower steady-state currents (150–165 A vs. 180 A), improving efficiency in high-altitude conditions.

#### 5.2.3. Normalized Thrust Step-Response Curves at 4000 m

Figure 5b overlays normalized thrust step-responses at 4000 m. PID: 18% overshoot, 0.6 s rise, 1.3 s settling. Lookup: 10% overshoot, 0.3 s rise, 1.0 s settling. HAARN: 3.5% overshoot, 0.3 s rise, 0.6 s settling. HAARN provides faster, more stable regulation in thin air.

#### 5.2.4. Motor Temperature During Continuous Hovering at 4500 m

Figure 5c shows motor temperature over 30-min maximum-load hovering at 4500 m and 5 °C. PID exceeds thermal threshold, shutting down at 10 min. Lookup approaches unsafe levels. HAARN stabilizes at 80 °C, staying below threshold, enabling sustained operation without damage.

#### 5.2.5. Schematic of the Test Setup

Figure 5d diagrams the test rig, including octocopter UAV with PT1000 sensors, environmental sensors (barometer, GPS, IMU), vacuum pump, six-axis load cell, tether, cold air circulation, and real-time data logging. This setup replicates high-altitude conditions for accurate performance measurements.

### 5.3. Adaptability and Energy Consumption Synergy Analysis

This section is devoted to the analysis of the synergistic optimization effect of HAARN in terms of energy efficiency ETE and thermal reliability.

#### 5.3.1. Energy Efficiency Comparison Analysis

We compared ETEs of the three methods at different altitudes and demonstrated that a higher ETE indicates stronger endurance. In flight tests, ETE is computed by integrating instantaneous battery power P=V·I and actual thrust *T*.

#### 5.3.2. ETE Comparison at Different Altitudes

**Figure 6:**
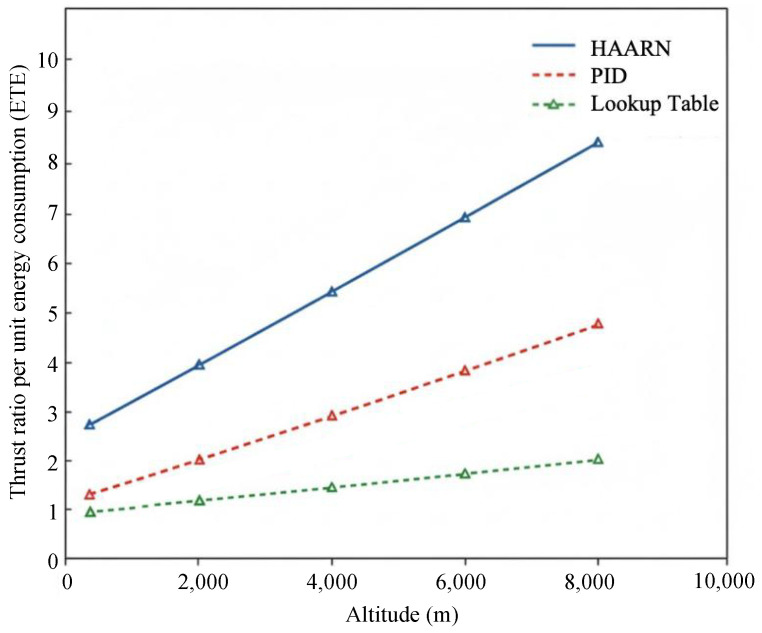
Comparison results of thrust ratio (ETE) per unit of energy consumption at different altitudes.

As indicated in Figure 6, the HAARN approach has the highest ETE at all altitudes. At an altitude of 4000 m, HAARN is about 8.3% higher compared to the simple lookup table method and 15.2% higher compared to traditional PID. This confirms that HAARN can achieve an optimal balance between thrust compensation and energy consumption management using the loss function $L_{ETE}$ during the training process, thus prolonging the endurance time of heavy-duty UAVs at high altitudes.

#### 5.3.3. Dynamic Thermal Management and Reliability

Analysis We further tested the thermal management performance of the system under continuous hovering conditions at an extreme altitude of 4500 m with maximum load. The test lasted 30 min with the motor surface temperature $T_{surface}$ reaching 90°C as the thermal safety threshold.

As shown in Table 3, due to dynamic current limit $I_{limit}$, HAARN maintains the peak motor temperature at 81°C, far below the upper threshold 90°C, without triggering any thermal protection in 30 min. By contrast, the conventional PID controller triggered a shutdown protection due to an excessive rise in temperature in 15.5 min, where the peak temperature increased to 98°C. The lowest rate of temperature increase is 2.5°C/min as HAARN effectively tunes an active thermal management strategy through the penalty term $L_{Heat}$. In addition to achieving the highest energy efficiency, HAARN has the longest continuous operating time under extreme conditions, significantly improving the operational reliability and safety of heavy-duty UAVs during plateau UHV tasks.

### 5.4. Model Quantization Evaluation

To evaluate the effect of the FP32-to-INT8 quantization on system performance, we compare the thrust compensation accuracy, thermal behavior, and control dynamics before and after quantization. As shown in Table 4, the INT8 model preserves the performance of the FP32 model with negligible differences. The thrust loss rate, ETE, overshoot, and peak motor temperature remain almost unchanged, while the model size is reduced by more than 70% and the inference latency improves from 35 ms to 7 ms, ensuring real-time execution on embedded hardware.

## 6. Conclusions

In this work, we proposed HAARN, a lightweight deep neural network–based regulation method for the high-altitude adaptability of heavy-load UAV power systems. By fusing multi-sensor environmental and system-state inputs and optimizing for thrust-per-unit-energy (ETE) under thermal constraints, HAARN reduced thrust attenuation to 2.5% at 4000 m and achieved energy-efficiency improvements of 8.3–15.2% compared to baseline methods across tested altitudes. The model’s dynamic current-limiting strategy also improved thermal reliability, enabling continuous hovering at extreme altitudes without triggering thermal protection.

Future work will focus on extending HAARN to online adaptive learning (e.g., integration with DRL) to support rapidly changing environments, expanding evaluation to additional UAV platforms and longer endurance tests, and integrating transient meteorological factors such as wind gusts into the regulation framework. We will also release anonymized dataset summaries and inference-code benchmarks to aid reproducibility.

## Figures and Tables

**Table 1:** Performance comparison of different regulation methods at 4000 m altitude.

Regulation Method	Target Thrust Loss Rate (%)	Average Motor Speed (rpm)	Average Current Consumption (A)	Regulation Response Time (ms)
Traditional PID [12]	15.0	4800	12.5	150
Simple Lookup Table [18]	9.8	5100	14.2	100
HAARN (Proposed)	2.5	5050	13.0	40

**Table 2:** Dynamic performance comparison of different regulation methods at 4000 m altitude.

Regulation Method	Overshoot (%)	Settling Time (s)	Rise Time (s)	Dynamic Characteristic Summary
Traditional PID [12]	18.0	1.5	∼0.6	Large overshoot, long settling time, prone to attitude oscillation
Simple Lookup Table [18]	10.0	1.2	∼0.5	Reduced overshoot but with 3% low-frequency oscillation
HAARN (Proposed)	3.5	0.6	∼0.3	Minimal overshoot, short settling time, optimal dynamic response

**Table 3:** Comparison of average motor temperature rise and duration under high-altitude extreme load (4500 m).

Regulation Method	$T_{\mathrm{env}}$ (°C)	$T_{\mathrm{motor},\mathrm{peak}}$ (°C)	Continuous Hovering Time (min)	Average Temperature Rise Rate (°C/min)
Traditional PID [12]	5	98	15.5	5.9
Simple Lookup Table [18]	5	88	25.0	3.3
HAARN (Proposed)	5	81	>30.0	2.5

**Table 4:** Performance comparison before and after model quantization (4000 m altitude).

Performance Metric	FP32 Model	INT8 Quantized Model
Thrust Loss Rate (%)	2.4	2.5
ETE (Thrust per Unit Energy)	9.2	9.1
Regulation Response Time (ms)	38	40
Overshoot (%)	3.4	3.5
Motor Peak Temperature (4500 m, °C)	80	81
Model Size	1.2 MB	0.3 MB
Inference Speed (Single Sample)	35 ms	7 ms

## Data Availability

The data presented in this study are available on request from the corresponding author.

## Abbreviations

The following abbreviations are used in this manuscript:

UHV	Ultra-High Voltage
HD-UAVs	Heavy-Duty UAVs
DNN	Deep Neural Networks
HAARN	High-Altitude Adaptive Regulation Network
EKF	Extended Kalman Filter
ETE	Effective Thrust per Energy/Thrust per Unit Energy Consumption Ratio
ESC	Electronic Speed Controllers
ISA	International Standard Atmosphere
BLDC	Brushless Direct Current (Motor)
MCU	Microcontroller Unit
FPU	Floating-Point Unit
RMSE	Root Mean Square Error
